# Reality–fantasy collapse in schizophrenia vs. neurocognitive impairment during Rorschach’s III card responding

**DOI:** 10.1192/j.eurpsy.2021.1393

**Published:** 2021-08-13

**Authors:** I. Gornushenkov, I. Pluzhnikov, S. Sorokin

**Affiliations:** 1 Department Of Endogenous Mental Disorders And Affective States, The Mental Health Research Center, Moscow, Russian Federation; 2 Department Of Adult Neuropsychology And Abnormal Psychology, Moscow Institute of Psychoanalysis, Moscow, Russian Federation; 3 Department Of Youth Psychiatry, The Mental Health Research Center, Moscow, Russian Federation

**Keywords:** Reality-Fantasy Scale, Rorschach, schizophrénia, Neurodegenerative diseases

## Abstract

**Introduction:**

Adaptive thinking demands a balance between manifestations of intrapsychic activity and reliance on requirements of the outer reality. Features of responses to Rorschach’s III card could provide information about subject’s ability to preserve the dialectical tension between the two poles of external and internal realities during solving tasks related to interpersonal relationships.

**Objectives:**

To compare reality-fantasy relations during Rorschach’s III card responding in patients with schizophrenia, neurocognitive impairment and normal subjects.

**Methods:**

Participants were 12 young adult inpatients with schizophrenia, 14 students without mental disorders and 12 inpatients with neurodegenerative diseases of old age. Reality-Fantasy Scale (RFS) was applied to assess responses to Rorschach’s III card. RFS scale ranges from –5 (reality collapse into fantasy) to 5 (fantasy collapse into reality) (Tibon-Czopp et al., 2015).

**Results:**

Patients with schizophrenia (M= –3,38, SD= 1,9) demonstrated tendency to fantasy domination (and reality collapse) if compared with the students (M= –1,47, SD= 2,0, p<0,05). Patients with neurodegenerative diseases (M= 0,75, SD= 2,1), conversely, had difficulties to apply fantasy during solving Rorschach task (p<0,01).

**Conclusions:**

Express Rorschach testing using III card could be useful to provide screening data of thinking tendencies related to situations of social interaction. Also it provides a mental pabulum regarding role of cognitive impairment in schizophrenia in relation to significance of affective dependence of their thinking process.

**Conflict of interest:**

The reported study was funded by RFBR, project number 20-013-00772

